# Thinking Before Doing: A Pilot Study on the Application of Motor Imagery as a Learning Method During Physical Education Lesson in High School

**DOI:** 10.3389/fspor.2020.550744

**Published:** 2020-10-06

**Authors:** Patrizio Canepa, Antonella Sbragi, Filippo Saino, Monica Biggio, Marco Bove, Ambra Bisio

**Affiliations:** ^1^Department of Experimental Medicine, Section of Human Physiology, University of Genoa, Genoa, Italy; ^2^Department of Neuroscience, Rehabilitation, Ophthalmology, Genetics, Maternal and Child Health, University of Genoa, Genoa, Italy; ^3^Ospedale Policlinico San Martino, Istituto di Ricovero e Cura a Carattere Scientifico, Genova, Italy

**Keywords:** motor imagery, learning, physical education, high school, volleyball

## Abstract

Motor imagery (MI), i. e., the mental simulation of an action without its actual execution, is a promising technique to boost motor learning via physical practice in rehabilitation, sport, and educational fields. The purpose of the present pilot study was to test the feasibility and the effectiveness of the application of MI as learning methodology place alongside conventional teaching technique as employed for physical education lessons. Thirty-three high school students from two classes were enrolled for instruction in the underhand serve in volleyball. One group, the motor imagery group (MIG) carried out the physical exercise along with the kinesthetic MI of the action, while the other group (the control group) was limited to the merely physical exercise. The training period lasted 8 weeks. MI duration and the duration of real movement (ME), the isochrony index (differences between real and imagined movements duration), and the number of balls which passed over the net (NBN) were evaluated before and after training. Results showed a significant improvement in the isochrony index for the MIG group exclusively; namely, MI duration became more similar to ME duration. Moreover, in MIG a significantly negative relationship appeared between the percentage change in the isochrony index and the difference between NBN before and after training. These findings suggest improvement in sensorimotor representation of the action, which lies at the basis of enhanced motor performance. The present study constitutes initial proof of concept on the application of MI as learning technique applicable to physical education lesson at high school.

## Introduction

Motor imagery (MI) is the mental simulation of an action without its actual execution (Jeannerod, 2001). A large body of evidence supports the existence of a functional equivalence between MI and movement execution (ME) [see Bisio and Bove (2018)]. This equivalence appears when comparing the response of the autonomic system (Decety et al., 1993) but also cortical and sub-cortical brain activations (Fadiga et al., 1999; Guillot et al., 2014; Bonzano et al., 2016) during MI and ME. Furthermore, at behavioral level a number of studies have applied the *mental chronometry paradigm* and have shown that in the case of simple gestures the duration of real and imagined movements corresponded, in compliance with the *isochrony principle* (Decety and Michel, 1989; Decety et al., 1989; Bisio et al., 2014).

On these bases, motor imagery has been successfully applied as motor learning technique (Avanzino et al., 2015; Di Rienzo et al., 2016; Ruffino et al., 2017; Bisio and Bove, 2018; Bisio et al., 2018). Moreover, it has been suggested as promising add-on to physical therapy in facilitating motor recovery in neurological patients (Abbruzzese et al., 2016; Guerra et al., 2017; Hanson and Concialdi, 2019) and to the conventional training in different sport domains for improving motor performance (Guillot and Collet, 2008; Bisio and Bove, 2018).

Dedicated literature showing the beneficial effects of motor imagery on motor performance dates back to the mid-1930s (Sackett, 1934, 1935). In practice, routine imagery is a well-established technique among sport experts (Hall et al., 1990; Jones and Stuth, 1997). Several studies showed the effectiveness of MI in connection with movement execution in sports requiring individual performance involving strength and flexibility, not to mention activities such as tennis and golf (Yue and Cole, 1992; Brouziyne and Molinaro, 2005; Guillot et al., 2010; Lebon et al., 2010). MI potential was also shown in team sports, examples being volleyball, basketball, and soccer (Blair et al., 1993; Al-Abood et al., 2002; Weinberg et al., 2003; Seif-Barghi et al., 2012; Afrouzeh et al., 2015).

In addition, motor imagery has been applied as an education technique in the medical field. Medical and nursing students, as well as resident staff with limited experience in surgery, were recruited for studies testing the effect of MI on a medical serve. Schuster and colleagues (Schuster et al., 2011) summarized the results of these studies and concluded that MI is a valuable educational technique to be implemented as a method in the clinical domain—also proposed by a more recent review (Anton et al., 2017). Although in sports possessing a high level of experience seems to increase MI efficacy (Olsson and Nyberg, 2010), in education most studies were aimed at students with limited or no experience in the requested performance. The positive results obtained by the latter point to the possibility of adopting motor imagery as an educational tool in schools.

Given these positive results as reported in sports and education literatures, motor imagery could be hypothesized as an appropriate tool to increase learning effectiveness as part of a physical education lesson at school.

Furthermore, motor imagery technique complies properly with the European Council Recommendation (released on 22 May 2018, 2018/C 189/01) dealing with key competences for lifelong learning. One of the eight key competences is the “Personal, social and learning to learn competence,” which includes the ability to reflect upon oneself and effectively manage time and information, to identify one's capacities, to focus, and deal with complexity, and to reflect critically and make decisions. Motor imagery might promote the development of this ability since it helps people to concentrate on complex tasks and to find solutions to complete such tasks—taking both internal and environmental information as a starting point. A potential consequence, therefore, of the application of MI to a physical education lesson might be an improvement in self-awareness, as suggested by studies on body perception and body schema (Baccarini et al., 2014) and by works showing a superposition between the frontoparietal networks active during MI and supporting awareness (Schwoebel and Coslett, 2005; Calabrò et al., 2015; Koch et al., 2016). Despite such potential, as far as is known, no study has yet tested whether MI could be applied alongside motor practice in a school environment.

The aim of this pilot study has been to test the feasibility and effectiveness of the application of motor imagery as a learning method alongside conventional teaching techniques founded on verbal instruction and movement execution during physical education lessons at high school.

## Methods

### Participants

Fifty-four students in their first year at an Italian high school volunteered to participate in this study. They were divided in two classes of 26 (10 males and 16 females, mean age ± SD = 14.12 ± 0.33) and 28 students (9 males and 19 females, mean age ± SD = 14.19 ± 0.41). The first was designated as the Motor Imagery Group (MIG), the second one as the Control Group (CG). Written informed consent was obtained from all participants and legal guardians before data collection. The study was conducted in accordance with the Declaration of Helsinki and was approved by the Ethical Committee of the University of Genoa (Comitato Etico per la Ricerca di Ateneo, n° 2020.1).

### Experimental Procedure

**Figure 1:**
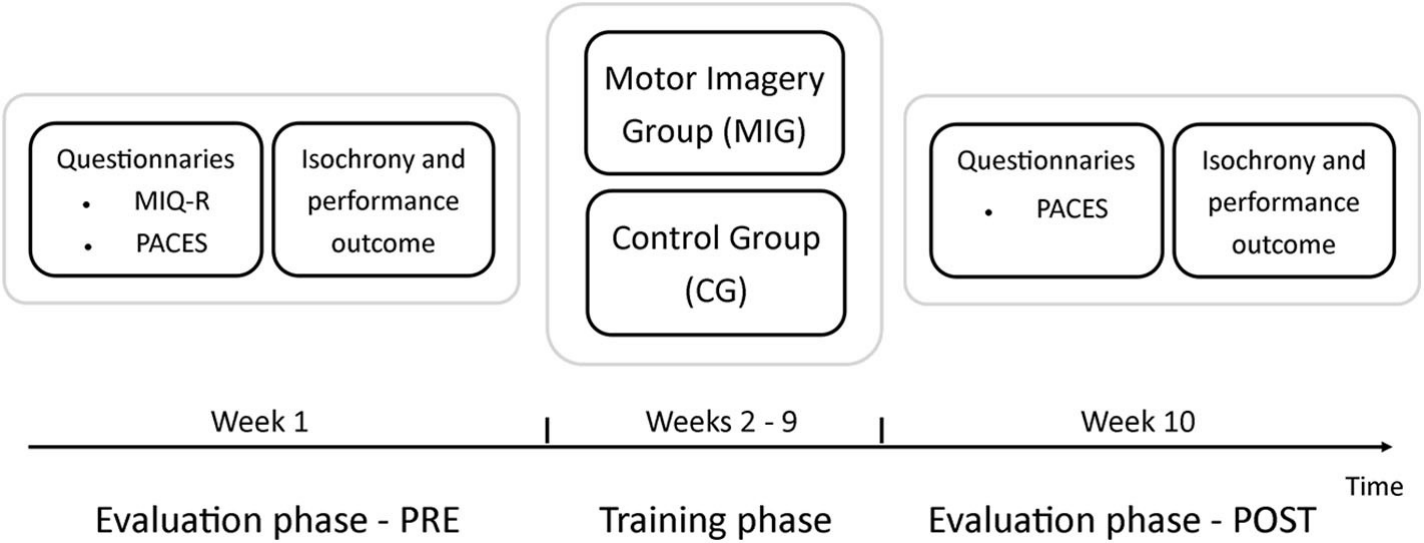
Illustration of experimental design: During the first week of the experimentation (Week 1), the experimenter performed the evaluation phase (PRE). Questionnaire on motor imagery ability (MIQ-R) and enjoyment of physical education lesson (PACES) were administered. After that, participants performed the underhand serve (3 times) and imagined the same gesture. The isochrony between imagined and real movement and the number of ball that passed over the net (performance outcome) were computed. From Week 2 to Week 9, the two classes either performed the conventional training based on movement execution (Control Group) or added to the conventional training a motor imagery training (Motor Imagery Group). During Week 10, the experimenter replicated the same tests administered during week 1 (POST).

The experiment lasted 10 weeks (Figure 1) and was effected as part of the physical education lesson, timetabled once a week. At the first lessons, a dedicated experimenter cooperated with the teacher to explain the aim of the project and performed baseline evaluations (PRE). From week 2 to week 9, students took part in the training sessions, scheduled for the first 15 min, and conducted by the experimenter in collaboration with the teacher. For the rest of the double period (1 h and 45 min), the teacher proceeded with his syllabuses, not comprising the topic of the experimentation. The tenth lesson was dedicated to the experimenter's evaluations (POST), enabling assessment of possible differences from PRE evaluations. Evaluation phases and training sessions took place in a gymnasium.

#### Evaluation Phase

Student assessment covered different kinds of evaluations, all of them conducted by the experimenter.

##### Questionnaires

In the beginning, each student received two questionnaires, each on a separate sheet. Students sat in a circle on the floor with the expert positioned in the center, from which position the first questionnaire instructions were read. The students were required to provide a written answer to each point of the questionnaire. Once they had completed the operation, the expert collected the papers with the answers to the first questionnaire and then proceeded in like manner for the second.

The first questionnaire was the Italian version of the Movement Imagery Questionnaire (MIQ-R) (Hall and Martin, 1997)—designed to evaluate the ability to form kinesthetic and visual images. MIQ-R is an 8-item self-report questionnaire, in which participants rated the vividness of their mental representations using two 7-point scales (associated with visual and kinesthetic imagery): 1 corresponds to “really easy to feel/see” whereas 7 corresponds to “really difficult to feel/see” (best score = 8, worst score = 56). Participants completed this questionnaire for PRE evaluation exclusively in order to assess their motor imagery ability—this latter considered good and comparable between groups (mean ± SD; MIG: 19±1.8; CG 19.5±1.8, *t* = *0.19, p* = *0.85*).

The second questionnaire was the Physical Activity Enjoyment Scale (PACES) [revised version by Motl et al. (2001)], modified and translated into Italian (Zocca et al., 2016), and specifically focused on physical education lesson—the aim being to test whether introducing the MI technique changed perceived enjoyment of the lesson. PACES comprises a 16-item scale that assesses enjoyment for physical activity by asking participants to rate “how you feel at the moment about the physical activity you have been doing” using a 5-point Likert scale, from 1 (I totally disagree) to 5 (I totally agree).

##### Isochrony and performance outcome

The objective of this test was to evaluate the correspondence between the duration of real and imagined movements and the number of balls that passed over the net, bouncing into the opposite court (NBN). Concerning movement duration, the hypothesis was that, the lower the difference between one duration and the other, the more imagined movement (motor imagery—MI) resembles the real movement (movement execution—ME). Then, an increase in NBN after the training period would be an index of enhanced performance.

At the beginning of this session, the teacher explained the basic technique of the underhand serve and provided a brief video tutorial. The video was the same for both classes. After the teacher's explanation, students were required to draw up so as to act in turns just beyond the edge of the volleyball court. Once the correct posture had been adopted, they had to carry out three underhand serves. The experimenter used a chronometer to measure the time lapse between the first movement the student started in preparation to serve and actual striking of the ball. The time taken and technical success of the serve (NBN) were recorded on paper. In the end, again in turns, each student was required to take up his position once again, and to imagine the same action three times. During MI, participants were required to perform a kinesthetic imagery [i.e., to focus on the sensory information generated during the actual action execution, including the strength and effort (Callow and Hardy, 2004)] of the action previously carried out. Further, they were instructed to say “*start*” when starting to imagine the movement and “*stop*” when their hand struck the ball virtually. The experimenter once again employed a chronometer for MI durations and took note accordingly. In the evaluation phase, participants were asked to focus exclusively on their own movement, and not on the ball movement, in order to create a comparison between imagined movement and real movement duration.

#### Training Session

##### Motor imagery group (MIG)

Students carried out three underhand serves during each session. They were positioned in three rows just beyond the edge of the volleyball court—in the left corner, the right corner, and the center. All at the same time, three students went through the procedure of imaginary and then real underhand serve. Once serving had been accomplished in their starting line, each student moved away so as to take up a position in one of the other lines. In this way, each student executed the movement in each of the three positions. Before engaging in any movement, they were instructed to firstly imagine the gesture. During training, the solution adopted was combined kinesthetic imagery, when the focus was on oneself, and visual imagery, when the focus was external on ball trajectory. When motor imagery technique is applied in the laboratory setting, it is common to focus on only one modality (Bisio et al., 2014; Avanzino et al., 2015; Bonassi et al., 2017). Conversely, during practical applications in sport the modalities, as well as the focus, are less specific—thus affording the athletes the possibility to pay attention to different aspects of the action (Blair et al., 1993; Brouziyne and Molinaro, 2005). The specific instruction was as follows: “*Imagine the underhand serve. Focus on the sensation you feel during the actual execution, and then visualize the ball that gets over the net and falls inside of the opposition court*.”

##### Control group (CG)

Students in the control group carried out the same training as those in the MIG—except for MI before the serving action. Typical learning methods during physical education lesson did not demand intervention other than the repetition of the action. This is why it was decided to not add placebo manipulation to conventional training—avoiding possible confusing effects.

### Data Analysis

The evaluation of the two types of training was performed by comparing the PACES score, the duration of real and imagined movements (mean values obtained over the 3 serves), and the NBN (computed as the sum of the 3 serves), before (PRE) and after (POST) training. The *isochrony index* (s) was calculated as the difference between the mean duration of imagined and real movement. A difference equal to zero yields perfect isochrony between the two movements.

Normality was checked by means of Shapiro–Wilk tests. PACES score, durations of real and imagined movements, and the isochrony index were normally distributed.

PACES score and the isochrony index were analyzed by means of ANOVAs with TIME (2 levels, PRE and POST) as a within-subject factor and GROUP (2 levels, MIG and CG) as a between-subject factor.

The statistical analysis on durations of real and imagined movements was performed by means of an ANOVA with TIME (2 levels, PRE, and POST) and MOVEMENT (2 levels, ME and MI) as within-subjects factors and GROUP (2 levels, MIG and CG) as a between-subject factor.

The NBN values were not normally distributed. In order to compare the two groups at PRE and POST Mann–Whitney tests were applied, while to test the difference between PRE and POST in each group Wilcoxon tests were used.

In both groups, Spearman's correlation was used to evaluate the relationship between the percentage changes in the isochrony index, computed as (isochrony index_POST_-isochrony index_PRE_)^*^100, and the differences between the number of ball that passed over the net (NBN_POST_-NBN_PRE_). The lower the isochrony percentage change, the better the improvement of the isochrony from PRE to POST. The higher (NBN_POST_-NBN_PRE_), the better the performance outcome from PRE to POST.

All the statistical analyses were performed by means of Statistica.

## Results

The experimentation comprised 54 students. Unfortunately, the analysis concerned only 32 students (16 in MIG and 16 in CG). Some students' data were removed because of absence from one of the evaluation phases; others were not considered because they participated in fewer than 6 lessons out of 8. Power of sample was calculated using *a posteriori* analysis by means of G-Power software, which showed good results for isochrony index, with η^2^ = 0.17 (see results of ANOVA on isochrony index), effect size = 0.45, 1–β = 0.99.

Mean values of all parameters are reported in Table 1.

**Table 1:** Mean values (± standard error) of parameters used to evaluate changes before (PRE) and after (POST) the training in the motor imagery group (MIG), who added motor imagery (MI) to movement execution (ME), and in the control group (CG), who performed only ME.

	**MIG**		**CG**	
	**Pre**	**Post**	**Pre**	**Post**
PACES score	3 ± 0.1	3.06 ± 0.1	2.91 ± 0.08	3 ± 0.1
**Movement duration (s)**				
ME	0.52 ± 0.03	0.6 ± 0.04	0.65 ± 0.06	0.61 ± 0.05
MI	1.69 ± 0.14	1.62 ± 0.14	1.85 ± 0.19	1.89 ± 0.15
Isochrony index (s)	1.17 ± 0.13	1.02 ± 0.14	1.20 ± 0.19	1.28 ± 0.15
NBN	1.94 ± 0.28	1.90 ± 0.27	1.94 ± 0.27	1.88 ± 0.24

*NBN, number of balls that passed over the net*.

The statistical analyses on PACES score revealed neither significant differences between the two evaluation epochs and between the two groups nor any significant interaction between TIME and GROUP.

**Figure 2:**
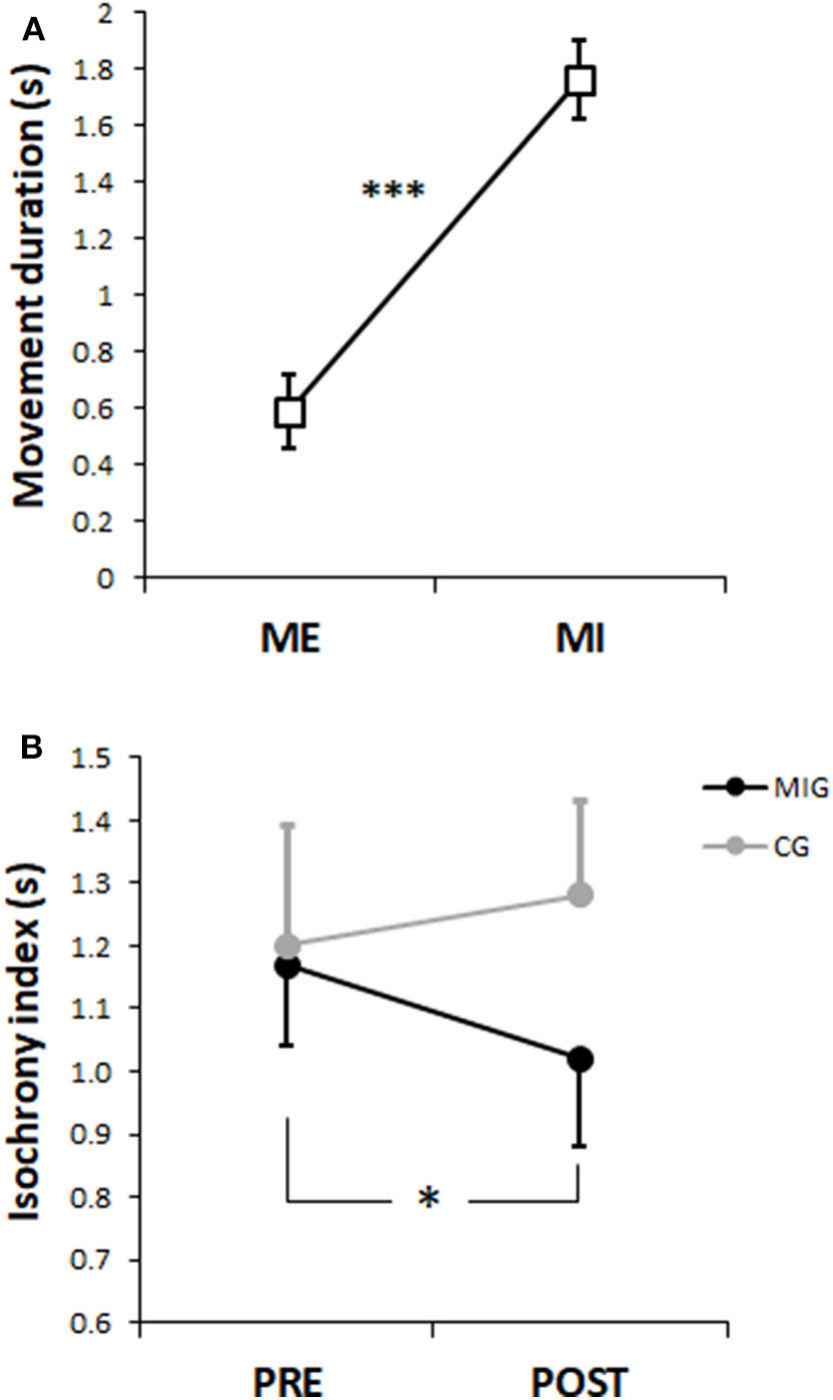
**(A)** Mean values of the duration (s) of executed (ME) and imagined (MI) movements. **(B)** Isochrony index mean values of the Motor Imagery Group (MIG—black circles) and Control Group (CG—gray circles). Error bars indicates standard errors. *p < 0.05, ***p < 0.001.

The comparison between real and imagined movement duration showed a significant effect of the factor MOVEMENT [F(1, 30) = 115.67, *p* < 0.0001, η^2^ = 0.79], indicating a significantly higher duration of the MI vs. ME (Figure 2A).

The analysis on the isochrony index showed a significant interaction between TIME and GROUP [F(1, 30) = 6.26, *p* < 0.05, η^2^ = 0.17). The Newman–Keuls *post hoc* comparisons revealed a significant decrease in the isochrony index value of MIG after the training period (*p* = 0.02; Figure 2B).

The analysis on NBN did not show any significant difference between groups both at PRE and at POST, nor were there any significant changes between PRE and POST in the two groups.

**Figure 3:**
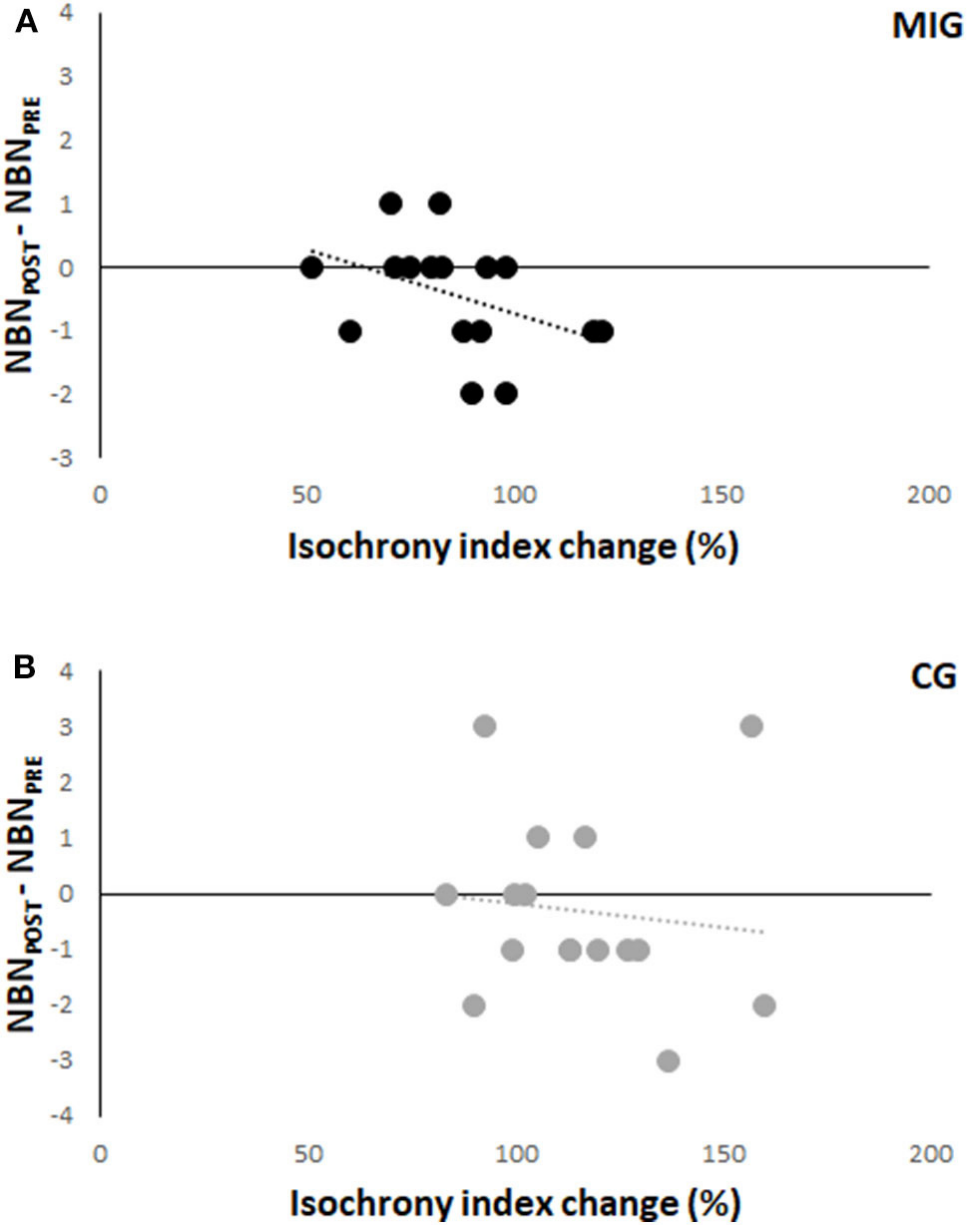
Relationship between the difference from baseline to after the training period in the number of ball that passed over the net (NBN_POST_-NBN_PRE_, y-axis), and the percentage changes in isochrony index (x-axis) in Motor Imagery Group [MIG, **(A)**] and Control Group [CG, **(B)**]. Each circle represents the data of a one participant.

Spearman's correlation between (NBN_POST_-NBN_PRE_) and the isochrony percentage change was significant for MIG (R = −0.50, *p* = 0.046) and showed that participants who improved NBN after MI training were those who showed a higher isochrony after the training than before. No significant correlation was observed in CG (R = −0.26, *p* = 0.33; Figure 3).

## Discussion

The aim of this pilot study was to test the feasibility and the effectiveness of a new teaching program that combines movement execution with motor imagery, as applied to physical education lesson in a high school context.

The results showed no differences between motor imagery and control groups on perceived enjoyment of the lesson (PACES score). Imagined movement duration was significantly longer than that of real movement—irrespective of the group and evaluation epochs. Furthermore, the isochrony index—a parameter yielding information on similarity between imagined and real movement—was computed. The results showed a significant decrease in index value after the training period exclusively in the motor imagery group, indicating that the duration of the imagined movement approached the duration of the real movement in MIG but not in CG. As regards performance index, the number of balls that passed over the net was counted. No changes appeared in this parameter in either group after the training. A significantly negative relationship appeared between the percentage change in the isochrony index and the differences between NBN from baseline (PRE) to the end of the training period (POST).

The application of motor imagery has by this stage become widespread in neuroscientific literature, where the majority of studies published on this matter show an improved motor performance after training based on MI (for instance Driskell et al., 1994; Gentili et al., 2006, 2010; Gentili and Papaxanthis, 2015). The improvements did not focus merely on behavioral response but also on plastic modifications to the cerebral activity, similar to those to be observed after physical practice (Pascual-Leone et al., 1995; Avanzino et al., 2015).

In the field of sport, and specifically in volleyball, Afrouzeh et al. (2015) showed that volleyball beginners, who combined physical practice and the PETTLEP model (i.e., a model providing a framework for the effective execution of motor imagery use), improved their “passing” abilities more than the other groups. Here, the focus has been on the MI effect on another gesture in volleyball—the underhand serve, one of the first basic skills acquired by beginners. Motor imagery technique is often used by elite athletes [70–90% reporting they use MI for improving their performance (Hall et al., 1990; Jones and Stuth, 1997)], who mastered the technical skills of their sport. Nevertheless, beginners too might benefit from such application, as shown in the seminal study on piano performance by Pascual-Leone et al. (1995) and in studies published in educational literature (Schuster et al., 2011). However, the lack of experience in volleyball might explain the lack of isochrony between real and imagined movements; the duration of imagined movements was significantly higher than that of real movements in both groups and testing epochs. Nor was this the first study showing the absence of isochrony between real and imagined movements. In a study on motor imagery performance in tennis players and gymnasts, Guillot et al. (Guillot et al., 2004) found that the duration of the imagined movements was higher than the case of executed movements. In order to explain this result, the explanation was offered that image accuracy would appear to be a more important factor than the temporal characteristics of the movement, leading to more time dedicated to imagining the action than to executing the same. In the present study, the increase in time devoted to MI vs. ME might have been caused by the difficulty the participants experienced in creating a kinesthetic imagine of this new action. Participants, therefore, might have needed more time to imagine the action accurately. Nevertheless, whether the expertise level alters/facilitates the temporal equivalence between executed and imagined movement duration remains to be determined in future studies (Guillot et al., 2012). Another factor that might have altered the isochrony is the actual time of the day (the morning) when the test and the training took place. It was shown that isochrony changed throughout the day, depending on the circadian rhythm—maximum between 2 and 6 p.m. (Gueugneau et al., 2009). Unfortunately, the high school timetable had already been established before experiment inception and it was not possible to alter it.

In face of this result, however, the present study showed that the difference between imagined and real movements, as it appears on the isochrony index, evolved differently in the two groups. In the control group, no differences emerged before and after conventional training, whereas in the motor imagery group the value decreased significantly after protocol administration. Changes in isochrony index might indirectly point to possible changes in the cortical sensorimotor representation of the movement. For this reason, a suggestion is that improvement in isochrony might be a consequence of an improved ability to gather the sensory information required to plan the movement. Such information might comprise visual information concerning the volleyball court, proprioceptive information concerning the posture the student has to adopt in the different phases of the action, and the strength the student has to channel to the ball so that it might pass over the net, at the same time respecting the area of the opposing court. This improvement might also result from facilitation in creating the motor plan to complete the movement. These results are signs of the occurrence of motor learning and are at the basis of a better motor performance. The significantly negative relationship found exclusively in the motor imagery group between improvements in isochrony and in performance before and after training supports this hypothesis. Participants showing improvements in isochrony after MI training (isochrony index % change <100) were students showing higher success in performing underhand serves (NBN > 0) or students who had not worsened (NBN = 0). Improvements in the mental representation of the movement, therefore, enhanced/preserved the effectiveness of the volleyball serve. However, it has to be acknowledged that motor imagery might have been a strong source of motivation for the experimental group, leading its participants to achieve better concentration during testing procedure. The improvements in isochrony, thus, might not be ascribed merely to the neurophysiological effects of motor imagery but also its motivational role.

No improvements whatsoever were recorded in NBN—considered as an index of performance. An explanation might lie in the limitations of the present study. A possible drawback was the limited time devoted to the experiment (about 15 min per lesson for a total of 8 weeks), structured to interfere as little as possible with the activity already planned for the physical education lesson proper. Such a time span might not have been sufficient to improve the motor performance. Indeed, it is worth noting that improvements occurred neither in the control nor in the motor imagery groups. To highlight such an issue, it would have been useful to devise a questionnaire at the end of the training period to ascertain how training was executed and how was participants' experience about that. Answers would have helped to infer possible criticisms explaining the lack of skill enhancement.

In specific reference to the role of motor imagery, the following hypothesis cannot be underestimated; lack of volleyball experience might have decreased the quality of the imagined movement and, consequently, its effectiveness. Furthermore, MI quality might have suffered because of the time of day training was scheduled, as has already been mentioned before.

Future studies dealing with this matter might, therefore, be organized so that motor imagery testing and training sessions are held in the afternoon and along with motor imagery as a learning method employed throughout the entire lesson.

Finally, a possible variability was introduced by the experimenter with chronometer employment to assess movement duration. Since this study was a preliminary test to assess the feasibility of the application of this procedure at school, the same study had to make do with the technological means at the school's disposal.

In conclusion, the present pilot study is the first to test the feasibility and the effectiveness of the application of motor imagery as add-on technique to conventional learning methods for physical education lesson at high school. As regards feasibility, the students positively adhered to the program and were curious about this new method. The majority of the participants from the motor imagery group anecdotally reported that they learnt to concentrate explicitly on the action and to imagine the same before accomplishing. This paper's proposal is that future experimentations might be designed so as to integrate motor imagery throughout the entire lesson and for a longer period of time, thus further assessing its potential in performance enhancement. Despite the abovementioned limitations, results are encouraging in terms of the improved sensorimotor ability shown by the motor imagery group and not by the control group. The conclusion is that this study embodies the first proof of concept aimed at introducing motor imagery as educational method for schools and might be considered an example in didactic innovation.

## Data Availability Statement

The raw data supporting the conclusions of this article will be made available by the authors, without undue reservation.

## Ethics Statement

The studies involving human participants were reviewed and approved by Comitato Etico per la Ricerca di Ateneo, University of Genoa (n° 2020.1). Written informed consent to participate in this study was provided by the participants' legal guardian/next of kin.

## Author Contributions

AB and PC conceived the idea of the study. AB, AS, and MBo designed the experiment. FS and PC carried out the experiments. AB, PC, MBi, and MBo were involved in the analysis and interpretation of data for the work. AB and PC drafted the manuscript. AS, FS, MBi, and MBo carried out the critical revision of the manuscript. All the authors approved the final version for publication, agreed to be accountable for all aspects of the work in ensuring that questions related to the accuracy or integrity of any part of the work are appropriately investigated and resolved, and made substantial contributions to the conception and design of the work.

## Conflict of Interest

The authors declare that the research was conducted in the absence of any commercial or financial relationships that could be construed as a potential conflict of interest.
